# Microstructure and First Hydrogenation Properties of TiHfZrNb_1−x_V_1+x_ Alloy for x = 0, 0.1, 0.2, 0.4, 0.6 and 1

**DOI:** 10.3390/molecules27031054

**Published:** 2022-02-04

**Authors:** Salma Sleiman, Jacques Huot

**Affiliations:** Hydrogen Research Institute, Université du Québec à Trois-Rivières, 3351 des Forges, Trois-Rivières, QC G9A 5H7, Canada; salma.sleiman@irh.ca

**Keywords:** metal hydrides, high entropy alloys, BCC, V substitution, kinetics

## Abstract

The effect of the substitution of Nb by V on the microstructure and hydrogen storage properties of TiHfZrNb_1−x_V_1+x_ alloys (x = 0.1, 0.2, 0.4, 0.6 and 1) was investigated. For x = 0, the alloy was pure BCC and upon the substitution of niobium by vanadium, the BCC was progressively replaced by HCP and FCC phases. For x = 0.6, a C15 phase was also present and becomes the main phase for x = 1. The substitution greatly enhanced the first hydrogenation and makes it possible at room temperature under 20 bars of hydrogen. The capacity of all substituted alloys was around 2 wt.%.

## 1. Introduction

Metal hydrides are known as one of the most practical solutions of hydrogen storage as they store hydrogen safely with high volumetric density under mild temperature and pressure [1]. Recently, high entropy alloys (HEAs) have attracted attention as novel materials for hydrogen storage. HEAs are multiple principal elements as described by Cantor et al. [2] and Yeh et al. [3]. The random distribution of the elements in HEAs provides a large diversity of local environments for hydrogen. The vastness of compositions in HEAs offers a large opportunity to tune their properties to practical applications [4,5].

One of the earliest studies of HEA-based metal hydrides was conducted by Kao and al. [6]. They carried out a systematic investigation of the effects of different x, y, and z values on the hydrogen properties of CoFeMnTi_x_V_y_Zr_z_ alloys (0.5 ≤ x ≤ 2, 0.4 ≤ y ≤ 3.0, and 0.4 ≤ z ≤ 3.0). All samples had a C14 Laves phase structure and could absorb hydrogen at room temperature after a prior heat treatment. After heat treatment at 400 °C, the CoFeMnTiVZr_2.3_ alloy had a maximum capacity of 1.79 wt.% at room temperature under 9.8 bars of hydrogen. The hydrogen absorption kinetics was greatly enhanced for the alloys with higher amounts of Ti and Zr. This enhancement was explained by the large atomic radii of Ti and Zr compared to that of V. Adding more Ti or Zr enlarges the size of the interstitial sites, and in turn, the lattice expansion that facilitates the hydrogen diffusion [6]. Chen et al. investigated the C14-Cr_u_Fe_v_Mn_w_Ti_x_V_y_Zr_z_ alloys [7]. A heat treatment at 400 °C was performed before the kinetic measurements. The kinetic measurements were conducted for each composition at different temperatures of 5, 25, and 80 °C under 9.8 bars of hydrogen. The Fe-free alloy CrMnTiVZr showed the highest maximum capacity of 2.23 wt.% at 5 °C [7]. The alloys V_35_Ti_30_Cr_25_Mn_10_, V_35_Ti_30_Cr_25_Fe_5_Mn_5_, and V_35_Ti_30_Cr_25_Fe_10_ were studied by Liu et al. [8]. The samples had mainly a body-centered cubic (BCC) structure. The V_35_Ti_30_Cr_25_Mn_10_ readily absorbed hydrogen at room temperature after heating at 100 °C, but the other alloys showed an incubation time [8]. Yang et al. systematically studied the (VFe)_60_(TiCrCo)_40−x_Zr_x_ alloys for x = 0, 1, and 2 [9]. All alloys were able to absorb hydrogen at room temperature, reaching a full capacity of 3.5 wt.% in 15 min, without prior heat treatment [9]. Sahlberg et al. showed that the BCC TiVZrNbHf alloy has a hydrogen storage capacity of 2.5 H/M (2.7 wt.%) at 300 °C [10]. Such capacity was explained by hydrogen occupancy in both tetrahedral and octahedral sites in the fully hydrided body centered tetragonal (BCT) structure [10]. Ek et al. investigated 21 alloys with compositions related to TiVZrNbHf [11]. After heating at 340 °C for 2 h in a dynamic vacuum, most of these alloys absorbed hydrogen at room temperature under 40 bars of pressure, reaching a full capacity of H/M = 2. However, when V was partially or completely removed, the kinetics became too slow. After several days of hydrogen exposure of 40 bars at room temperature, TiV_0.5_ZrNbHf reached only H/M of 1.3. TiZrNbHf, TiZrNb, TiNbHf, TiZrHf, and ZrNbHf alloys required heating at 500 °C instead of 340 °C and hydrogenation at 300 °C instead of room temperature [11]. We see that, in all these previous investigations, a heat treatment was needed before the first hydrogenation. However, for practical purposes, it would be preferable to perform the first hydrogenation at room temperature.

Recently, we investigated the effect of the particle size, hydrogenation temperature, and hydrogenation pressure on the kinetics absorption of the TiVZrNbHf alloy [12]. The sieving of particles to a size less than 0.5 mm made the alloy absorb hydrogen below 300 °C. At 200 °C, a long incubation period of 27 h was needed before the alloy started to absorb hydrogen. However, once started, the hydrogenation was relatively fast, reaching a 2.2 wt.% (2.0 H/ M) capacity. In another work, we selected the transition elements of period 4 to synthesize the Ti_0.3_V_0.3_Mn_0.2_Fe_0.1_Ni_0.1_ alloy [13]. Two synthesis methods were used: arc melting and ball milling. Irrespective of the synthesis method, the alloy absorbed hydrogen at room temperature under 20 bars of hydrogen without any prior heat treatment.

In this study, we substituted Nb by V in the TiHfZrNb_1−x_V_1+x_ alloy. The reason for this substitution is that V is lighter than Nb. Thus, the hydride could potentially have a higher gravimetric hydrogen storage capacity. Here, we report the investigation of the crystal structure and hydrogen storage properties of TiHfZrNb_1−x_V_1+x_ alloys (x = 0.1, 0.2, 0.4, 0.6, and 1).

For each alloy, we calculated the thermodynamic, geometric, and electronic parameters for phase formation rules. The thermodynamic parameter is characterized by the ratio of the entropy of mixing over the enthalpy of mixing (Ω = T ΔS_mix_/ΔH_mix_) of the alloys. The geometric parameter δ is a function of the atomic size difference of the constituent elements. The electronic parameter VEC gives the valence electron concentration of the alloy. The expression for each parameter is given in [14]. Table 1 lists the values of ΔS_mix_, ΔH_mix_, Ω, and δ for each investigated alloy. The value of VEC was 4.4 for all investigated alloys.

We can see that ΔS_mix_ decreased with x, reaching the lowest value for the Nb-free (x = 1) alloy. ΔH_mix_ and Ω also decreased with x and their values satisfied the conditions for the formation of solid solution phases. In the case of δ, it increased with x and was the maximum for x = 1. VEC was 4.4 for all alloys and this value favors the formation of the BCC structure over the face-centered cubic (FCC) structure [16]. According to the Ω and VEC values, all the selected compositions fulfilled the conditions for the formation of the BCC phase. However, referring to δ values, the formation of intermetallic compounds is expected.

## 2. Results and Discussions

### 2.1. Microstructural Study

**Figure 1:**
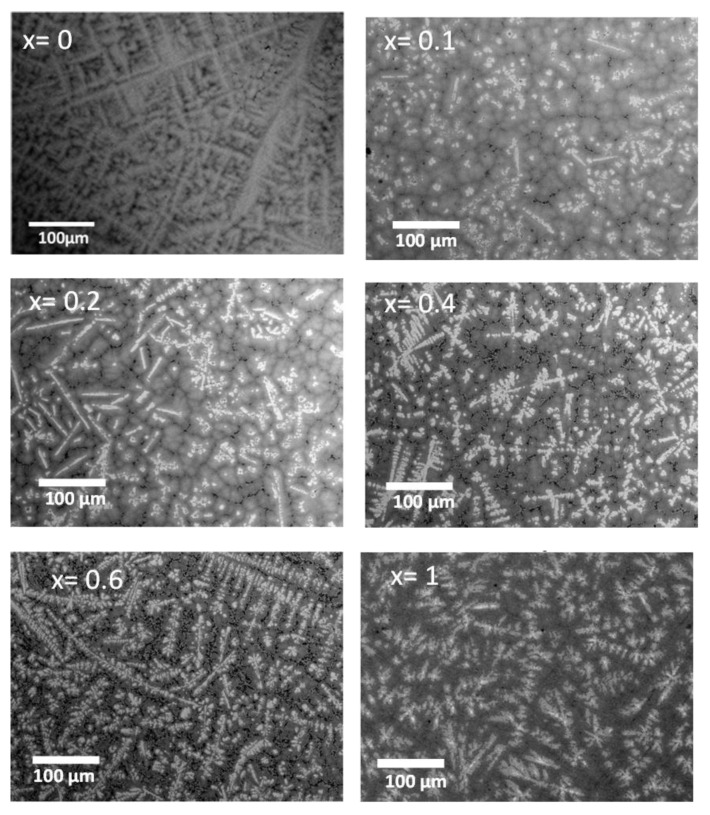
Backscattered electron (BSE) micrograph of the TiHfZrNb_1−x_V_1+x_ alloys for x = 0, 0.1, 0.2, 0.4, 0.6, and 1.

Figure 1 shows the backscattered electron micrograph of TiHfZrNb_1−x_V_1+x_ alloys for x = 0, 0.1, 0.2, 0.4, 0.6, and 1.

The bulk chemical composition for each sample was confirmed by EDX measurements to be equal to the nominal values. From Figure 1, it is clear that all compositions had a dendritic structure, but the microstructure changed with x. The TiHfZrNbV alloy (x = 0) was made of light grey dendrites surrounded by darker grey regions and a few darker (almost black) spots. A slight substitution of Nb by V (x = 0.1) made the dendrites much brighter, but also smaller. All the substituted alloys were found to be multiphase, showing a matrix with bright and dark phases. For total substitution (x = 1), the dark phase disappeared.

**Figure 2:**
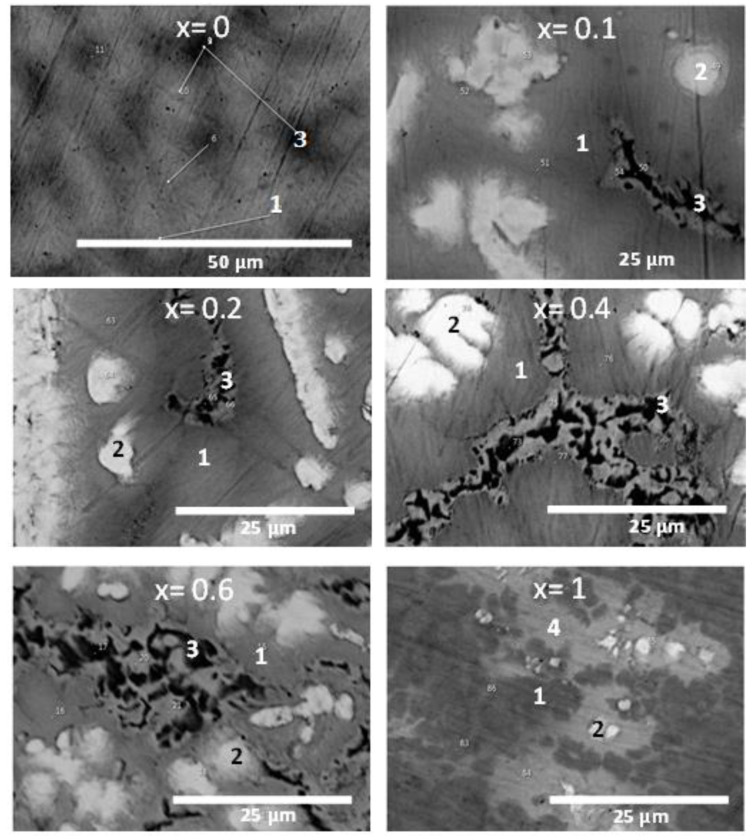
BSE micrographs of the TiHfZrNb_1−x_V_1+x_ alloys for x = 0, 0.1, 0.2, 0.4, 0.6, and 1 with higher magnification. x = 0 is from [12] with permission.

Using EDX at a higher magnification, the chemical compositions of the individual phases were measured on the selected points presented in Figure 2.

In a previous investigation, it was shown that the equiatomic sample TiHfZrNbV is a single phase BCC with a range of composition. This variation of composition is the origin of the change in grey shade seen in Figure 2 for x = 0 [12].

The other micrographs show that the alloys are made of a main phase, thereafter, called the matrix and indicated by point number 1, a bright phase (point 2), and a dark phase (point 3). For x = 1, no black region was present, but there was a bright grey phase (point 4). The chemical compositions of points 1, 2, and 3 are listed in Table 2, Table 3, and Table 4, respectively.

From Table 2, it can be seen that for x = 0, the matrix had a composition very close to the nominal one. For x = 0.1, the composition was again very close to the nominal one. For x = 0.2, the composition was still very similar to the composition of x = 0 and 0.1. For x = 0.4, we could see an important increase in vanadium content and a corresponding decrease in niobium. The other concentrations of the elements were still close to nominal. At x = 0.6, the matrix showed a strong depletion of niobium and an increase in titanium when compared to x = 0.4. Finally, for x = 1, the vanadium content was much higher than the nominal one and the titanium content was lower. From this, we can see that substitution of Nb by V does not result in a smooth variation of the matrix composition. Vanadium seems to be more abundant, while titanium was less abundant than what was expected from a linear variation of the element’s concentration with x.

The atomic compositions of the bright phase (point 2) are listed in Table 3. It is clear that the bright phase (point 2) was rich in Hf and Zr and depleted in Nb and V. From x = 0.1 to 0.6, the composition was almost constant, but there was a slight decrease in Nb proportion and a slight increase in Zr proportion, keeping the Zr + Nb proportion constant. For x = 1, the proportion of vanadium was much higher than for the other compositions.

The atomic compositions of the dark phase (point 3) are presented in Table 4. This phase was not present for x = 1. We could see that the dark phase was V-rich and its composition was nearly constant for all values of x.

For the bright grey phase (point 4, only for x = 1) of Figure 2, its atomic composition was 26Ti–24Hf–26Zr–24V, which was very close to the equiatomic composition.

### 2.2. Crystal Structure

**Figure 3:**
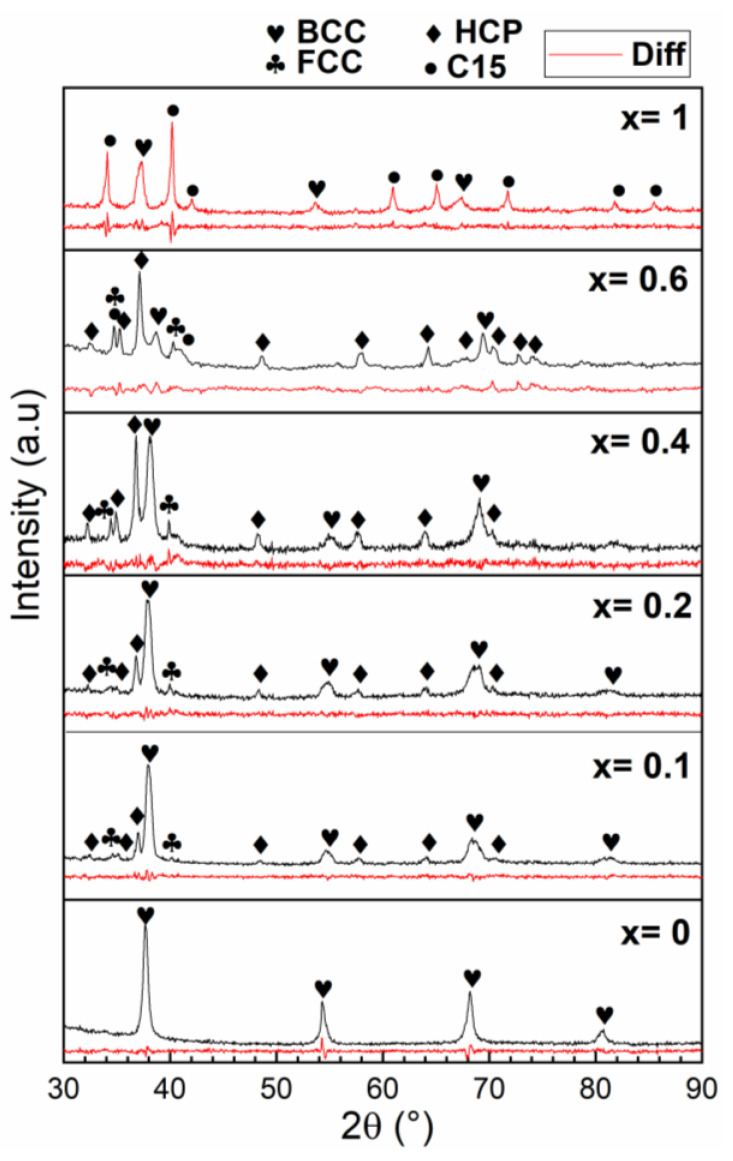
X-ray diffraction patterns of the as-cast TiHfZrNb_1−x_V_1+x_ alloys for x = 0, 0.1, 0.2, 0.4, 0.6, and 1. The bottom (red) line of each pattern is the residue of Rietveld refinement.

Figure 3 presents the XRD patterns of all compositions in their as-cast state.

For x = 0, the crystal structure was pure BCC. Other phases appeared upon the substitution. The abundance of each phase as determined by Rietveld refinement is reported in Table 5. For x = 0.1, 0.2, and 0.6, the main phase was the BCC phase along with a HCP phase and a minor amount of a FCC phase. As x increased from 0.1 to 0.6, the abundance of the BCC phase decreased, the HCP phase increased, and the FCC phase had roughly the same abundance. At x = 0.6, a C15 Laves phase appeared. For complete substitution of Nb by V, C15 becomes the main phase along a BCC phase.

The crystal structures seen in the diffraction patterns could be correlated to the chemical compositions of phases given by EDX. For x = 0.1 to 0.6, we could associate the matrix (point 1) to the BCC crystal structure.

The bright phase (point 2) could be associated with the HCP crystal structure for x= 0.1 to 0.6. From the chemical composition of the bright phase shown in Table 3, we could infer that the structure type of this HCP phase was Hf_0.5_Zr_0.5_. The phase diagram of Hf–Zr indicates that these two elements are totally miscible and have a HCP structure. The bright phase seen in the present samples was mainly made of hafnium (41% on average) and zirconium (38% on average). Therefore, the bright phase could be associated with a HCP phase of the chemical composition 0.14Ti–0.41Hf–0.38Zr–0.04Nb–0.02V.

For the patterns x = 0.1 to 0.6, a FCC phase was identified. From Figure 2, we can see that the dark phase is the only possibility of matching this phase. From Table 4, the average composition of the dark phase was 0.15Ti–0.07Hf–0.08Zr–0.1Nb–0.6V.

For x = 0.6 and 1, a C15 phase was identified in the diffraction pattern. In the case of x = 0.6, the only possibility was to assume that some of the dark regions in the micrographs are associated with this C15 phase and some others to a FCC phase. In fact, the x = 0.6 composition of the dark phase was 0.17Ti–0.08Hf–0.08Zr–0.08Nb–0.59V. This is very close to the composition of the dark phase for x = 0.1 to 0.4, but here, the atomic abundance of group 5 atoms (V and Nb) was exactly 0.67, while the total abundance of group 4 atoms (Ti, Zr, and Hf) was 0.33. The C15 phase had a structure of AB_2_, where A is a hydride forming element and B is a non-hydride forming element. Thus, we can assign this phase to a C15 structure (space group Fd-3m) where Ti/Zr/Hf are assigned to the 8b site (A atoms) and the B atoms V/Nb are on the 16c site. It is known that Laves phases are related to BCC. For example, Hao et al. have shown by molecular dynamics that a perfect C15 cluster could be embedded in BCC iron [17].

For x = 1, the situation is more complex. The C15 phase is the most abundant phase in the diffraction pattern. Compared to Figure 2, it is most likely to be the matrix with a composition of 0.12Ti–0.18Hf–0.16Zr–0.54V that could be written as Ti_0.36_Hf_0.54_Zr_0.48_V_1.62_. This is relatively far from an AB_2_ stoichiometry as most likely Ti, Zr, and Hf share the same site. The bright grey phase (point 4) seen in Figure 2 is associated with the BCC phase. The stoichiometry of that phase was 26Ti–0.24Hf–0.26Zr–0.24V.

The crystal structure parameters of the BCC phase as determined by Rietveld refinement are tabulated in Table 6 with the average atomic radius of this phase. The chemical composition of the BCC phase was taken from the EDX measurements for each x. The BCC phase is the matrix for x = 0.1 to 0.6 and the bright grey phase for x = 1.

The chemical composition of the BCC phase changes with x, but not in a linear fashion. However, from Table 6, we can see that the ratio of the average radius over the lattice parameter was practically constant.

The crystal structure parameters of the HCP phase are presented in Table 7. The lattice parameters of all samples were roughly constant with x. This is expected because, as shown in Table 3, the chemical composition for the HCP phase was almost the same for all samples.

The crystal structure parameters of the FCC phase are shown in Table 8. The lattice parameter of the FCC phase was almost the same for all compositions. This is consistent with the fact that the chemical composition of this phase is constant, as shown in Table 4. For this phase, the microstrain was refined, but the results were always zero within the experimental error.

The crystal structure parameters of the C15 phase are shown in Table 9. The lattice parameter of C15 phase increased with x. However, the main differences were the crystallite size and microstrain. For x = 0.6, only the microstrain could be refined. All attempts to refine the crystallite size provided unrealistic numbers. This is similar to the one encountered for another AB_2_ alloy [18]. It has been shown that when crystallite size is impossible to refine, the microstrain reflects a variation of chemical composition. This is also most likely the case here. Because the chemical composition varies within the dark phase, the crystal may adopt the FCC or the C15 structure. It should be pointed out that in the Rietveld refinement of the C15 phase for x = 1, the occupancy factor of vanadium was refined. The refined occupancy was 0.84, which translates to an abundance of 55 at.%. This value was very close to the measured value of 54 at.% in Table 2. This means that the C15 phase of x = 1 has an important number of vacancies on the B site.

### 2.3. First Hydrogenation

**Figure 4:**
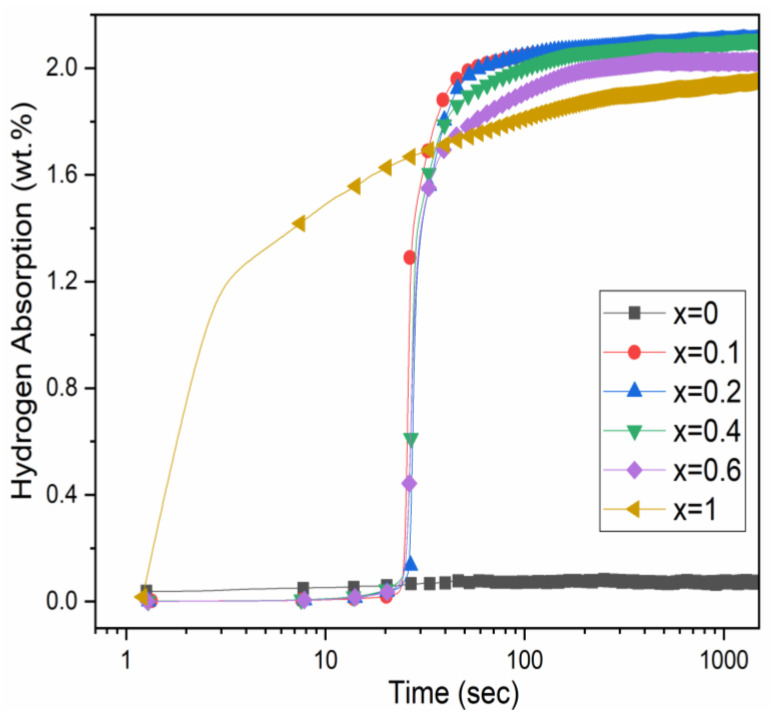
Activation curves of the TiHfZrNb_1−x_V_1+x_ alloys for x = 0, 0.1, 0.2, 0.4, 0.6, and 1.

The first hydrogenation (activation) of the TiHfZrNb_1−x_V_1+x_ alloys for x = 0, 0.1, 0.2, 0.4, 0.6, and 1 was performed at room temperature under a hydrogen pressure of 20 bars without any prior heat treatment. Results are presented in Figure 4.

The first hydrogenation for x = 0 is impossible at room temperature [10,12]. Substituting niobium by vanadium provided a good hydrogen uptake with 25 s of incubation time and fast kinetics. Surprisingly, even if the heavy element Nb was replaced by the lighter element V, there was a slight downward trend for the capacity with increasing x. Additionally, the incubation time and kinetics were essentially the same for all substituted alloys. The TiHfZrV_2_ alloy did not show any incubation time. The disappearance of the incubation time could be related to the predominant C15 phase.

**Figure 5:**
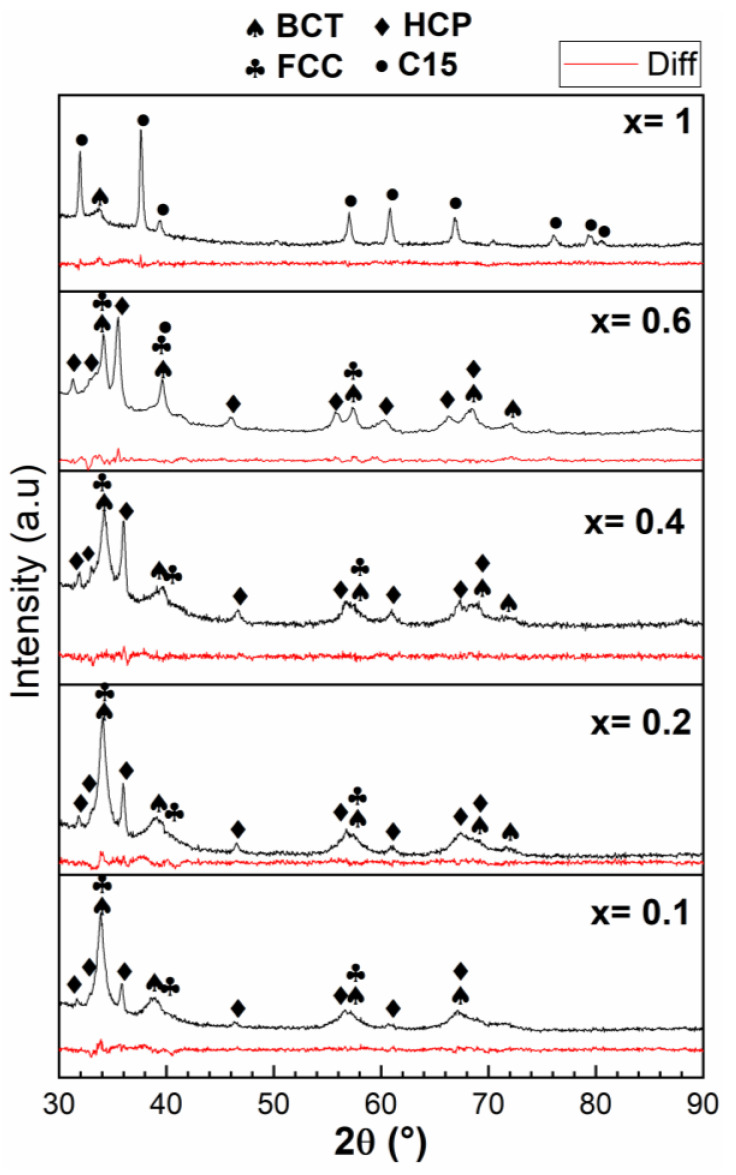
XRD patterns of the TiHfZrNb_1−x_V_1+x_ alloys in the hydrogenated state for x = 0.1, 0.2, 0.4, 0.6, and 1. The bottom (red) line of each pattern is the residue of Rietveld refinement.

The crystal structure of the hydrided alloys were investigated by XRD. The results are shown in Figure 5.

**Figure 6:**
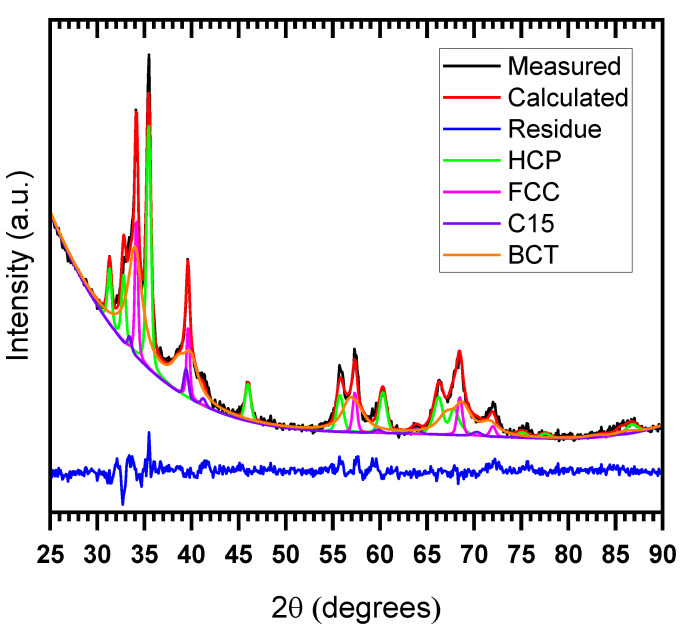
Rietveld refinement details of the XRD pattern of the hydrogenatedTiHfZrNb_0.4_V_1.6_ alloy.

For x = 0.1, 0.2, 0.4, and 0.6, the crystal structure was essentially BCT with HCP and FCC as a secondary phase. It has been reported that BCT is the structure adopted by a fully hydrided BCC high entropy alloy [10,12]. For the x = 1 sample, the main phase was C15 and BCT was the minor phase. The crystal structure parameters and the abundance of each phase in all hydrogenated samples as determined by Rietveld’s analysis are presented in Table 10. To show the relative contribution of each phase to the pattern, Figure 6 shows the details of the pattern with the most phases present (x = 0.6).

Basically, the same phases were present in the hydrided and as-cast patterns. From Table 10 and referring to Table 5, we can see that for x = 0.1 to 0.6, except for x = 0.4, the abundance of the FCC phase in the hydrogenated samples was higher than the abundance in the as-cast samples. For x = 0.4, the abundance of the FCC phase in the hydrogenated sample had relatively the same abundance as in the as-cast sample. From x = 0.1 to 0.2, the abundance of the HCP phase in the hydrogenated samples was close to the abundance in the as-cast samples. However, the HCP phase was less abundant in the hydrogenated samples for x = 0.4 and 0.6. The BCT phase abundance was relatively the same abundance as the BCC phase for x = 0.1 and 0.2. However, its abundance was higher than the BCC abundance for x= 0.4 and 0.6. Regarding the C15 phase for x = 0.6, the amount of the hydrided C15 phase was much lower than that in the as-cast (4% vs. 16%).

In the case of x = 1, we can see that the hydrogenated alloy had a 31 wt.% BCT phase, which corresponds to the abundance of the BCC phase (31%) in the as-cast alloy. Therefore, BCC was completely transformed into BCT upon hydrogenation. For the C15 phase, similar to the as-cast state, the occupancy factor of the B site corresponded to a vanadium abundance of 57 at.%.

Concerning the HCP phase and referring to Table 7, we can see that the crystallite size of this phase decreased upon hydrogenation for x = 0.1 to 0.4, but it increased for x = 0.6. No microstrains were found in the hydrogenated samples of x= 0.1 to 0.4, while a microstrain of 0.29% appeared for x = 0.6. For the C15 phase and using Table 9, the crystallite size in the hydrided C15 was greater than that of the as-cast when x = 1.

As the unit cell volume of all phases in the hydrided state was larger than in the as-cast state, we could use the volume increase to estimate the hydrogen capacity. Considering that a hydrogen atom produces a volume expansion between 2 and 3 Å^3^, the estimated amount of hydrogen in each phase was determined from the volume expansion ΔV of each hydrided phase. The results are shown in Table 11.

Taking into consideration the abundance of each phase in the hydrided samples, the estimated amount of hydrogen is shown in Table 12.

This table clearly shows that the measured capacities of all samples were in the middle of the estimated range. We could estimate that, on average, a hydrogen atom takes a volume of 2.7 Å^3^. This is very close to the volume expansion of estimation of 2.9 Å^3^ that Peisl found for a wide range of materials [19]. Using 2.7 Å^3^ for the hydrogen volume in the crystal structure, we estimated the H/M ratio for each phase. We found that the BCT phase had a ratio close to 2, and the HCP phase had a ratio between 0.75 and 1. Surprisingly, the FCC H/M ratio for x = 0.1, 0.2, 0.4, and 0.6 was 0.39, 0.28, 0.24 and 0.18, respectively. We also observed that the FCC phase took up less and less hydrogen as x value increased. Regarding the C15 phase, for x = 0.6, the H/M was 0.57 while for x = 1, the H/M was 1.25.

For x = 0.1 to 0.6, most of the capacity came from the BCT phase. This is because the estimated amount of hydrogen in the hydride BCT was higher than that of the hydride HCP and FCC phases and also because BCT was the most abundant over the hydrided phases. For x = 1, the estimated amount of hydrogen in the hydride C15 phase was higher than that of BCT. Additionally, C15 had the highest abundance (69%) compared to BCT (31%). Therefore, most of the measured capacity came from the C15 phase. Referring to the activation curves of Figure 4, one can correlate the absence of the incubation time to the presence of the C15 phase as the main phase.

**Figure 7:**
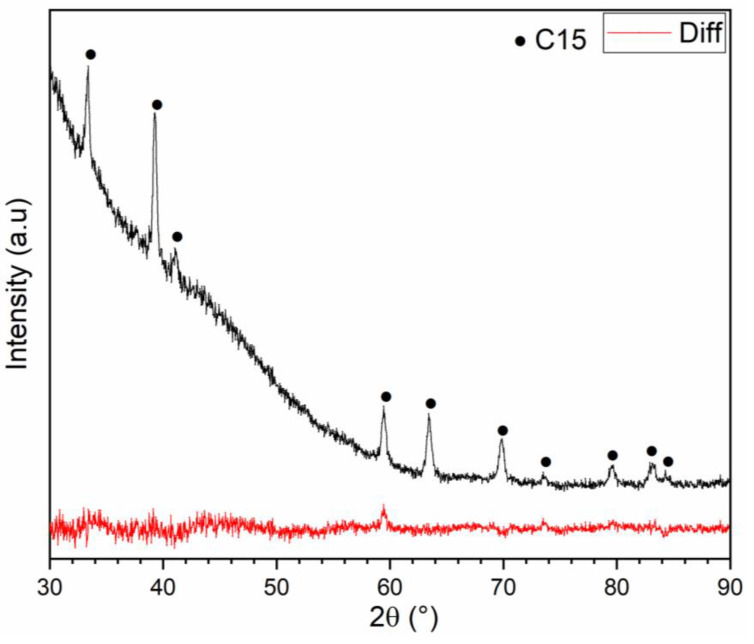
XRD pattern of the TiHfZrV_2_ alloy (x = 1) dehydrided at 400 °C under vacuum.

The TiHfZrV_2_ alloy was selected to study its dehydrided state. The choice of this composition is because it is the total substituted sample, and its synthesis is relatively easy compared to the other samples. However, studying the stability of the other hydrided compositions is planned for a future paper. After reaching full hydrogenation, the sample was subjected to vacuum for two hours at 350 °C. XRD measurements (not shown) confirmed that no desorption occurred. Thus, the temperature was raised to 400 °C and the sample kept under dynamic vacuum for two hours. The XRD pattern of the dehydrogenated sample is presented in Figure 7. The very high background is due to a special sample holder that kept the powder under an argon atmosphere.

We could see that only the C15 phase was present in the desorbed patterns; no BCC or BCT phases were seen. The reason is that the BCC/BCT phases were most probably ‘buried’ under the high background. The volume of the C15 phase as determined by Rietveld refinement was 443.8 Å^3^. The volume of the C15 phase after dehydrogenation was larger than that of the C15 phase in the as-cast state (412.1 Å^3^). This indicates that there is still hydrogen in the C15 phase. Assuming a volume of hydrogen atom of 2.7 Å^3^, the hydrogen still present is 0.73 wt.% (0.49 H/M). This means that the sample is not fully desorbed even at 400 °C under vacuum. This could be explained by the high stability of the binary hydrides of the raw elements where vanadium has the highest plateau pressure in the range 0 < H/M < 1 of about 10 kPa. A temperature higher than 400 °C is needed for full desorption, but this is over the limit of our apparatus.

## 3. Materials and Methods

All the raw materials, Ti sponge (99.95%), Hf sponge (99.6%), Zr sponge (99.5%), Nb pieces (99.8%), and V pieces (99.7%) were purchased from Alfa Aesar (Tewksbury,, USA) and used as received. After mixing all raw elements in the desired proportion, each alloy was prepared by arc melting under 0.7 bars of argon. Each pellet was melted, turned over, and remelted three times to ensure good homogeneity. Before the hydrogenation measurements, the as-cast alloy was hand-crushed under an argon atmosphere using a hardened steel mortar and pestle. The first hydrogenation of all alloys was measured at room temperature under 20 bar hydrogen pressure on a homemade Sievert’s apparatus. The powder was filled in the reactor and kept under dynamic vacuum for one hour at room temperature before the measurement. The crystal structure was determined by X-ray diffraction using a Bruker D8 Focus with Cu Kα radiation. Lattice parameters were evaluated from Rietveld refinement using Topas software [20]. Microstructure and chemical analysis were performed using a Hitachi Su1510 scanning electron microscope (SEM) (Hitachi, Mississauga, Canada) equipped with an EDX (energy dispersive X-ray) apparatus from Oxford Instruments (Abingdon, UK).

## 4. Conclusions

The effect of the substitution of Nb by V on the microstructure and hydrogen storage properties of the TiHfZrNb_1−x_V_1+x_ alloy for x = 0, 0.1, 0.2, 0.4, 0.6, and 1 was investigated. For x = 0, the alloy was pure BCC. All alloys with substitutions were multiphase. Upon substitution of niobium by vanadium, the BCC was progressively replaced with HCP and FCC phases. For high values of x, a C15 phase was present and became the main phase for x = 1. We found that substitution of Nb by V greatly enhanced the first hydrogenation and made it possible at room temperature under 20 bars of hydrogen. The BCC phase transformed to BCT in the fully hydrided state. For hydrogen storage purposes, the optimum amount of substitution seems to be the total substitution where there is no Nb in the alloy.

## Figures and Tables

**Table 1:** The parameters ΔS_mix_, ΔH_mix,_ Ω, and δ for the TiHfZrNb_1−x_V_1+x_ alloys (x = 0, 0.1, 0.2, 0.4, 0.6, and 1).

x Value	ΔS_mix_ J.K^−1^mol^−1^	ΔH_mix_ kJ.mol^−1^	Ω	δ %
Criteria for solid solution [15]	Maximum	−11 to 5	≥1.1	≤6.6
0	13.38	0.16	192	6.86
0.1	13.37	−0.13	235	6.99
0.2	13.31	−0.40	76	7.13
0.4	13.10	−0.97	30	7.39
0.6	12.74	−1.51	19	7.64
1	11.08	−2.56	9	8.08

**Table 2:** EDX analysis showing the elemental composition of the matrix (point 1) of the TiHfZrNb_1−x_V_1+x_ alloys for x = 0, 0.1, 0.2, 0.4, 0.6, and 1. Error on all values was 1 at.%.

x	Ti (at.%)	Hf (at.%)	Zr (at.%)	Nb (at.%)	V (at.%)
0	20	19	19	22	20
0.1	21	18	18	20	23
0.2	21	18	18	21	22
0.4	20	17	19	14	30
0.6	23	18	20	9	30
1	12	18	16	0	54

**Table 3:** EDX analysis showing the elemental composition of the bright phase (point 2) of: TiHfZrNb_1−x_V_1+x_ alloys for x = 0.1, 0.2, 0.4, 0.6, and 1. Error on all values was 1 at.%.

x	Ti (at.%)	Hf (at.%)	Zr (at.%)	Nb (at.%)	V (at.%)
0.1	15	42	36	5	2
0.2	15	40	38	5	2
0.4	14	43	37	4	2
0.6	14	41	41	2	2
1	13	38	36	0	13

**Table 4:** EDX analysis showing the elemental composition of the dark phase (point 3) of: HfZrNb_1−x_V_1+x_ alloys for x = 0.1, 0.2, 0.4, and 0.6. Error on all values was 1 at.%.

x	Ti (at.%)	Hf (at.%)	Zr (at.%)	Nb (at.%)	V (at.%)
0.1	14	6	7	12	61
0.2	14	7	10	12	57
0.4	14	7	8	9	62
0.6	17	8	8	8	59

**Table 5:** Abundance in wt.% of each phase in the TiHfZrNb_1−x_V_1+x_ alloys for x = 0, 0.1, 0.2, 0.4, 0.6, and 1. The number in parentheses is the error on the last significant digit.

x	0	0.1	0.2	0.4	0.6	1
BCC	100	80 (1)	76 (2)	59 (2)	38 (3)	31 (1)
HCP	…..	14 (1)	17 (1)	29 (2)	35 (3)	…..
FCC	…..	6 (1)	7 (1)	11 (1)	10 (1)	…..
C15	…..	…..	…..	…..	16 (2)	69 (1)

**Table 6:** Crystal structure parameters of the BCC phase in the TiHfZrNb_1−x_V_1+x_ alloys for x = 0, 0.1, 0.2, 0.4, 0.6, and 1. Error on the last significant digit is indicated in parentheses.

BCC	Cell Volume (Å^3^)	Lattice Parameter (Å)	Crystallite Size (nm)	Microstrain (%)	Average Radius (pm)	Ratio
0	37.99 (2)	3.362 (1)	9.9 (2)	1.01 (3)	147	2.29
0.1	37.92 (3)	3.360 (1)	30 (2)	0.31 (1)	146.1	2.30
0.2	37.51 (3)	3.347 (1)	25 (3)	0.35 (1)	146.2	2.29
0.4	37.01 (3)	3.333 (1)	10.5 (6)	0.23 (2)	145.5	2.29
0.6	36.79 (6)	3.326 (2)	4.1 (7)	………	145.9	2.28
1	39.81 (3)	3.415 (1)	25 (5)	0.38 (2)	148.5	2.30

**Table 7:** Crystal structure parameters of the HCP phase in the TiHfZrNb_1−x_V_1+x_ alloys for x = 0.1, 0.2, 0.4, and 0.6. Error on the last significant digit is indicated in parentheses.

HCP	Cell Volume (Å^3^)	Lattice Parameter (Å)	Crystallite Size (nm)	Microstrain (%)
0.1	45.57 (3)	a = 3.202 (1) c = 5.132 (2)	38 (9)	0.14 (2)
0.2	45.38 (3)	a = 3.199 (1) c = 5.121 (2)	41 (10)	0.13 (2)
0.4	45.46 (3)	a = 3.201 (1) c = 5.123 (2)	22 (2)	0.07 (2)
0.6	45.57 (3)	a = 3.203 (1) c = 5.129 (2)	21 (1)	……

**Table 8:** The crystal structure parameters of the FCC phase in the TiHfZrNb_1−x_V_1+x_ alloys for x = 0.1 to 0.6. Error on the last significant digit is indicated in parentheses.

FCC	Cell Volume (Å^3^)	Lattice Parameter (Å)	Crystallite Size (nm)
0.1	91.61 (1)	4.508 (3)	16 (3)
0.2	91.33 (1)	4.503 (2)	22 (5)
0.4	91.25 (1)	4.502 (2)	15 (2)
0.6	91.74 (6)	4.511 (1)	53 (17)

**Table 9:** Crystal structure parameters of the C15 phase in the TiHfZrNb_1−x_V_1+x_ alloys for x = 0.6 and 1. Error on the last significant digit is indicated in parentheses.

C15	Cell Volume (Å^3^)	Lattice Parameter (Å)	Crystallite Size (nm)	Microstrain (%)
0.6	397 (1)	7.350 (7)	…….	0.63 (4)
1	412.0 (2)	7.441 (1)	21.1 (6)	….

**Table 10:** The crystal parameters of each phase in the hydrogenated TiHfZrNb_1−x_V_1+x_ alloys for x = 0.1, 0.2, 0.4, 0.6, and 1. Error on the last significant digit is indicated in parentheses.

Sample	Phase	Cell Volume (Å^3^)	Lattice Parameter (Å)	Crystallite Size (nm)	Micro-Strain (%)	Abundance (%)
0.1	BCT	47.90 (6)	a = 3.286 (2) c = 4.437 (3)	8.1 (5)	0.48 (2)	76 (1)
	HCP	49.88 (6)	a = 3.255 (1) c = 5.435 (2)	15 (1)	……	11 (1)
	FCC	95.8 (2)	4.574 (2)	………	0.33 (2)	13 (1)
0.2	BCT	47.35 (5)	a = 3.273 (2) c = 4.419 (3)	7.5 (6)	0.49 (2)	73 (2)
	HCP	49.43 (5)	a = 3.246 (1) c = 5.418 (2)	17 (1)	……	14 (1)
	FCC	94.4 (1)	4.554 (2)	………	0.35 (2)	13 (1)
0.4	BCT	46.86 (8)	a = 3.265 (2) c = 4.395 (5)	5.2 (4)	0.60 (4)	65 (2)
	HCP	49.55 (4)	a = 3.249 (1) c = 5.421 (2)	15 (1)	………	22 (1)
	FCC	93.9 (1)	4.545 (2)	………	0.36 (2)	13 (1)
0.6	BCT	47.47 (9)	a = 3.193 (2) c = 4.657 (7)	3.3 (2)	………	57 (2)
	HCP	51.33 (4)	a = 3.296 (1) c = 5.456 (2)	36 (6)	0.29 (1)	24 (2)
	FCC	93.66 (8)	4.541 (1)	43 (20)	0.15 (2)	15 (2)
	C15	434 (1)	7.574 (6)	………	0.31 (4)	4 (1)
1	BCT	49.0 (2)	a = 3.339 (5) c = 4.40 (1)	7 (2)	1.09 (7)	31 (2)
	C15	492.8 (1)	7.899 (1)	31 (2)	0.07 (1)	69 (2)

**Table 11:** The variation of volume ΔV and the estimated range of hydrogen in each phase in the hydrogenated TiHfZrNb_1−x_V_1+x_ alloys for x= 0.1, 0.2, 0.4, 0.6, and 1.

	ΔV of BCT (Å^3^)	Estimated Amount of H in BCT (wt.%)	ΔV of HCP (Å^3^)	Estimated Amount of H in HCP (wt.%)	ΔV of FCC (Å^3^)	Estimated Amount of H in FCC (wt.%)	ΔV of C15 (Å^3^)	Estimated Amount of H in C15 (wt.%)
0.1	9.98	1.87 to 2.8	4.31	0.6 to 0.89	4.19	0.53 to 0.79	……	……
0.2	9.84	1.83 to 2.75	4.05	0.57 to 0.85	3.07	0.37 to 0.56	……	……
0.4	9.85	1.91 to 2.88	4.09	0.56 to 0.84	2.65	0.33 to 0.5	……	……
0.6	10.68	2.09 to 3.14	5.76	0.8 to 1.2	1.92	0.24 to 0.36	37	0.76 to 1.14
1	9.19	1.68 to 2.52	……	…………	……	……	80.8	1.7 to 2.55

**Table 12:** Estimated capacity of the phases in the hydrided samples.

	0.1	0.2	0.4	0.6	1
Estimated amount of H in the hydride (wt.%)	1.56 to 2.33	1.46 to 2.2	1.4 to 2.12	1.45 to 2.18	1.69 to 2.54
Measured capacity (wt.%)	2.1	2.1	2.1	2	1.95

## Data Availability

Data available by contacting the authors.

## Sample Availability

Samples of all the compounds are available from the authors.

## References

[1] Von Colbe J.B., Ares J.-R., Barale J., Baricco M., Buckley C., Capurso G., Gallandat N., Grant D.M., Guzik M.N., Jacob I. (2019). Application of hydrides in hydrogen storage and compression: Achievements, outlook and perspectives. Int. J. Hydrogen Energy.

[2] Cantor B., Chang I., Knight P., Vincent A. (2004). Microstructural development in equiatomic multicomponent alloys. Mater. Sci. Eng. A.

[3] Yeh J.W., Chen S.K., Lin S.J., Gan J.Y., Chin T.S., Shun T.T., Tsau C.H., Chang S.Y. (2004). Nanostructured high-entropy alloys with multiple principal elements: Novel alloy design concepts and outcomes. Adv. Eng. Mater..

[4] Ding Q., Zhang Y., Chen X., Fu X., Chen D., Chen S., Gu L., Wei F., Bei H., Gao Y. (2019). Tuning element distribution, structure and properties by composition in high-entropy alloys. Nature.

[5] Felderhoff M., Marques F., Balcerzak M., Winkelmann F., Zepon G. (2021). Review and outlook on high-entropy alloys for hydrogen storage. Energy Environ. Sci..

[6] Kao Y.-F., Chen S.-K., Sheu J.-H., Lin J.-T., Lin W.-E., Yeh J.-W., Lin S.-J., Liou T.-H., Wang C.-W. (2010). Hydrogen storage properties of multi-principal-component CoFeMnTixVyZrz alloys. Int. J. Hydrogen Energy.

[7] Chen S.-K., Lee P.-H., Lee H., Su H.-T. (2018). Hydrogen storage of C14-CruFevMnwTixVyZrz alloys. Mater. Chem. Phys..

[8] Liu J., Xu J., Sleiman S., Chen X., Zhu S., Cheng H., Huot J. (2021). Microstructure and hydrogen storage properties of Ti–V–Cr based BCC-type high entropy alloys. Int. J. Hydrogen Energy.

[9] Yang S., Yang F., Wu C., Chen Y., Mao Y., Luo L. (2016). Hydrogen storage and cyclic properties of (VFe)_60_(TiCrCo)_40−x_Zr_x_ (0 ≤ x ≤ 2) alloys. J. Alloys Compd..

[10] Sahlberg M., Karlsson D., Zlotea C., Jansson U. (2016). Superior hydrogen storage in high entropy alloys. Sci. Rep..

[11] Ek G., Nygård M.M., Pavan A.F., Montero J., Henry P.F., Sørby M.H., Witman M., Stavila V., Zlotea C., Hauback B.C. (2020). Elucidating the effects of the composition on hydrogen sorption in TiVZrNbHf-based high-entropy alloys. Inorg. Chem..

[12] Sleiman S., Huot J. (2021). Effect of particle size, pressure and temperature on the activation process of hydrogen absorption in TiVZrHfNb high entropy alloy. J. Alloys Compd..

[13] Sleiman S., Moussa M., Huot J. (2021). Microstructure and Hydrogen Storage Properties of the Multiphase Ti_0.3_V_0.3_Mn_0.2_Fe_0.1_Ni_0.1_ Alloy. Reactions.

[14] Zhang Y., Lu Z., Ma S., Liaw P., Tang Z., Cheng Y., Gao M. (2014). Guidelines in predicting phase formation of high-entropy alloys. MRS Commun..

[15] Zhang Y., Zhou Y.J., Lin J.P., Chen G.L., Liaw P.K. (2008). Solid-solution phase formation rules for multi-component alloys. Adv. Eng. Mater..

[16] Guo S., Ng C., Lu J., Liu C. (2011). Effect of valence electron concentration on stability of fcc or bcc phase in high entropy alloys. J. Appl. Phys..

[17] Wang H., Gao N., Lü G.-H., Yao Z.-W. (2018). Effects of temperature and point defects on the stability of C15 Laves phase in iron: A molecular dynamics investigation. Chin. Phys. B.

[18] Khajavi S., Rajabi M., Huot J. (2018). Crystal structure of as-cast and heat-treated Ti_0.5_Zr_0.5_(Mn_1−x_Fe_x_)Cr_1_, x = 0, 0.2, 0.4. J. Alloys Compd..

[19] Peisl H. (1978). Lattice strains due to hydrogen in metals. Hydrogen in Metals I.

[20] Bruker A. (2005). Topas V3: General Profile and structure ANALYSIS Software for Powder Diffraction Data—User’s Manual.

